# Predicting language diversity with complex networks

**DOI:** 10.1371/journal.pone.0196593

**Published:** 2018-04-27

**Authors:** Tomasz Raducha, Tomasz Gubiec

**Affiliations:** 1 Institute of Experimental Physics, Faculty of Physics, University of Warsaw, Pasteura 5, 02-093 Warsaw, Poland; 2 IFISC (CSIC-UIB), Instituto de Física Interdisciplinar y Sistemas Complejos, Campus Universitat de les Illes Balears, E-07122 Palma de Mallorca, Spain; 3 Center for Polymer Studies, Boston University, Boston, MA 02215 United States of America; Universidad Rey Juan Carlos, SPAIN

## Abstract

We analyze the model of social interactions with coevolution of the topology and states of the nodes. This model can be interpreted as a model of language change. We propose different rewiring mechanisms and perform numerical simulations for each. Obtained results are compared with the empirical data gathered from two online databases and anthropological study of Solomon Islands. We study the behavior of the number of languages for different system sizes and we find that only local rewiring, i.e. triadic closure, is capable of reproducing results for the empirical data in a qualitative manner. Furthermore, we cancel the contradiction between previous models and the Solomon Islands case. Our results demonstrate the importance of the topology of the network, and the rewiring mechanism in the process of language change.

## Introduction

Evolution and propagation of the world’s languages is a complex phenomenon, driven, to a large extent, by social interactions [1–3]. Multilingual society can be seen as a system of interacting agents [4–6], where the interaction leads to a modification of the language spoken by the individuals [7, 8]. Two people can reach the state of full linguistic compatibility due to the positive interactions, like transfer of loanwords. But, on the other hand, if they speak entirely different languages, they will separate from each other. These simple observations make the network science [9] the most suitable framework to describe and analyze dynamics of language change [10–12]. Although many mechanisms have been explained [13–16], we lack a qualitative description of the scaling behavior for different sizes of a population. Here we address the issue of the language diversity in societies of different sizes, and we show that local interactions are crucial to capture characteristics of the empirical data. We propose a model of social interactions, extending the idea from [17], that explains the growth of the language diversity with the size of a population of country or society. We argue that high clustering and network disintegration are the most important characteristics of models properly describing empirical data. Furthermore, we cancel the contradiction between previous models [18, 19] and the Solomon Islands case. Our results demonstrate the importance of the topology of the network, and the rewiring mechanism in the process of language change.

## Literature review

Language, as one of the most important aspects of our culture, has been studied using numerous different approaches [20]. Significant part of the research in quantitative linguistics applies methods or ideas taken from physics. For instance, already classical gravity model explaining language change and spatial diffusion [21, 22]. Also more recent works borrow tools from physical sciences, like agent based modeling [23], or scaling analysis [24]. Fokker-Planck equation approach has been used to simulate changes in language over time [25]. The term *statistical physics of language dynamics* is becoming more popular and this branch of science is already broadly developed [26, 27]. Research ranges from diffusion of modifications in spoken dialects [23, 28], to statistical and topological properties of written language [29, 30].

Diversity of language studies can be observed in the number of aspects analyzed. One important direction is geographical distribution and spatial diffusion in context of language change [31, 32]. On the other hand, properties of written texts are being explored in great detail [33–35]. From the former examples we can see, that complex networks are also applied in linguistic studies. And this framework has still more to offer besides the text analysis. Network is a natural representation of a society with a given topology of interactions represented by links. Therefore, it is straightforward to simulate new words creation, language change or competition on networks [6, 23, 26]. This approach is further justified by linguistics, as it states that language changes during human pair interactions [36].

Model analyzed in this paper deals with language change using coevolving complex networks [37] to reflect the society. We acknowledge that spatial distribution and geographical conditions are hugely important part of this subject [28, 31, 38]. Nevertheless, we want to understand other aspects of language change. We also omit creation of new words. In the model we have random initial conditions with a given set of dialect’s traits, which can evolve only through a copying mechanism. If two dialects of two individuals do not share any common traits, they cannot interact with each other and they destroy the connection. This process accounts the dynamics of real-world patterns of interaction. Additionally, complex networks can precisely describe interaction patterns in social media, which influence on the shape of languages is increasing [39].

In this work we use terms *language* and *dialect* separately in a sense of a dialect originating from a language. Although, the difference is not well defined and sometimes it is better to analyze just dialects [20, 40]. In general, speakers of different dialects of the same language should be able to communicate without a big effort. Measure of this kind of similarity is called mutual intelligibility. But this criterion is far from perfect, and can classify different languages as dialects of one language [41, 42]. Therefore, sociolinguistic context should also be considered. Nevertheless, we leave this subtle topic for linguists. In the work, we use data on languages and dialects. We do not want to justify the classification given in sources. A curious reader can do it himself, as all the data we analyze is cited and available.

## Results and discussion

**Fig 1:**
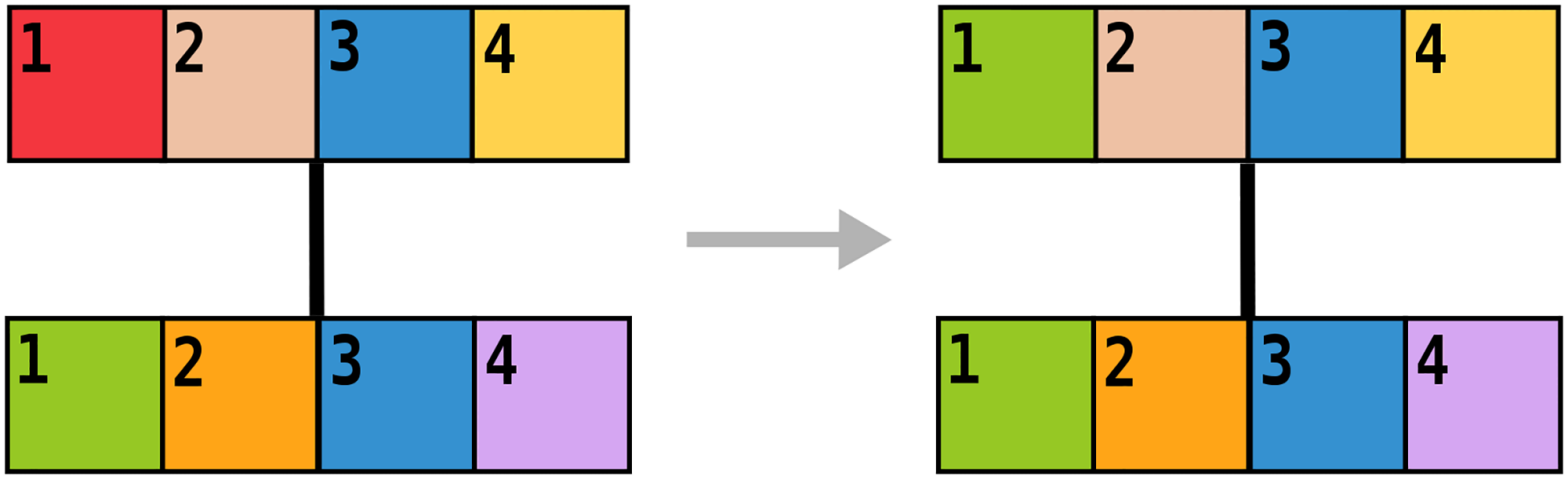
Schematic illustration of the positive interaction. Bars illustrate four traits (from 1 to 4, *F* = 4) of two interacting nodes. Every color is one of *q* possible values. The upper node is the active one. Left: before interaction only the trait number 3 has the same value (blue). Right: after interaction, in one of the possible realizations, the active node also adopts value of the first trait (green).

**Fig 2:**
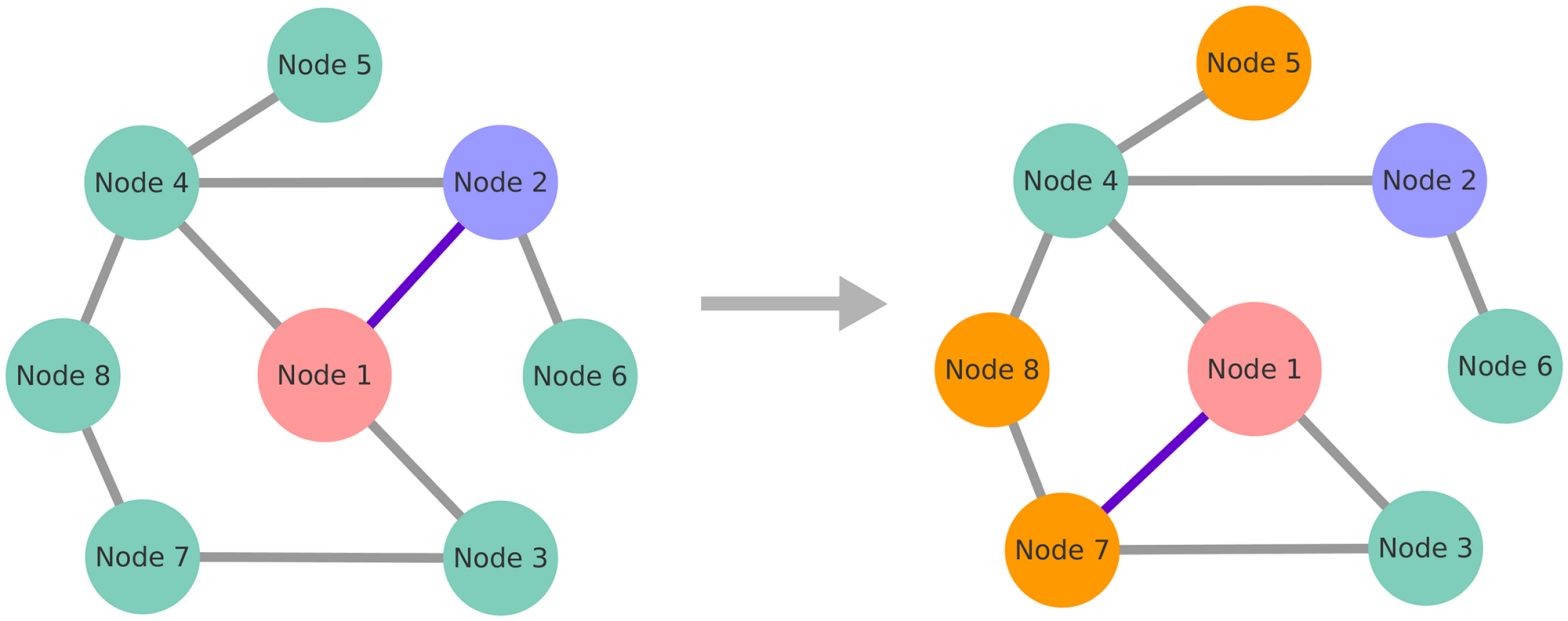
Schematic illustration of the rewiring mechanism. Node 1 (pink) is the active node. Left: consider its interaction with node 2 (purple). Assuming they have no common traits, the link between them must be rewired. Right: after erasing the edge, the active node can create a link to one of the nodes 5, 7, or 8 (orange ones). Node 7 is randomly selected.

Consider a system of *N* individuals, each using a language described by a set of *F* traits, similar to the models by Axelrod [18] and Schulze [11]. As in the Axelrod model, every individual has an associated vector *σ*_*i*_ = (*σ*_*i*,1_, *σ*_*i*,2_, …, *σ*_*i*,*F*_), where each entry can have one of *q* values *σ*_*i*, *f*_ ∈ {1, 2, …, *q*}, *f* = 1, 2, …, *F*. In Schultz model *q* = 2. Individuals are connected by links, indicating social interactions enabling language transfer. Two agents can speak very similar dialects or completely different languages, what is reflected in *q* different values of every trait. Traits should be interpreted as groups of words, or grammar rules, rather than single words. During the interaction people tend to adapt their languages to each other, if they have anything in common (see Fig 1). The more similar languages they speak, the more probable is the positive interaction and learning from each other, leading to a further increase of the similarity. On the other hand, people using languages with all traits different have no possibility to communicate and will cut the connection and look for a new neighbor. After disconnecting from a neighbor, active node will choose a new one from a set of vertices distant by two edges (see Fig 2), i.e. neighbors of neighbors. This type of rewiring is often called *triadic closure*. For detailed description of the dynamics see Materials and methods section. Note, that this copying mechanism is the same as in Axelrod and Schulze models, however they were defined on a static square lattice. Additionally, we do not consider random mutations, as in Schulze model.

This rewiring mechanism assumes only local interactions, what is intuitive for every-day use of language. It was shown that social networks are characterized by high value of the clustering coefficient [43–46]. This rewiring mechanism increases the value of the clustering coefficient by the definition. Additionally, triadic closure has been recognized as an important psychological and sociological mechanism [47, 48]. It has been also found in empirical studies on social networks [43, 49–51], thus we believe the proposed dynamics is in a good correspondence with real-world systems. Parameters *F* and *q* reflect the diversity of language. One trait can stand for a vocabulary in a given field. Than, different values of *q* indicate different words used to describe the same objects.

It was shown that the model defined as above displays three significantly different phases [17]. In the first phase, for small values of *q*, we observe death of most of the dialects. In this phase, when the system reaches the final configuration almost all agents speak the same language, and the graph is connected. In the second phase the network disintegrates into many small components, each with a different language. Society is polarized and different clusters use different languages. In the third phase a partial recombination occurs, but the number of languages increases further, resulting in existence of links between individuals speaking different languages. For that reason, the two first phases are more suitable for the explanation of the language change. Additionally, it is a reasonable assumption that languages can vary to a finite extent.

Despite the fact that this simple usage-based model of language manages to capture the essence of social interactions, its interpretation considering languages was abandoned after very first publication [18], due to the contradiction with the empirical data. Anthropological study of Solomon Islands in the late 70’s [52] showed that the number of languages functioning on an island grows with the size of the island. As noted in the original paper, results of the first model defined on a static square lattice were exactly opposite—the number of domains was decreasing with increasing size of the lattice. Moreover, the first adaptive model [19], taking into account coevolution of the nodes’ states and the topology of the network, did not solve this issue—the number of domains was approximately constant for different sizes of the network.

**Fig 3:**
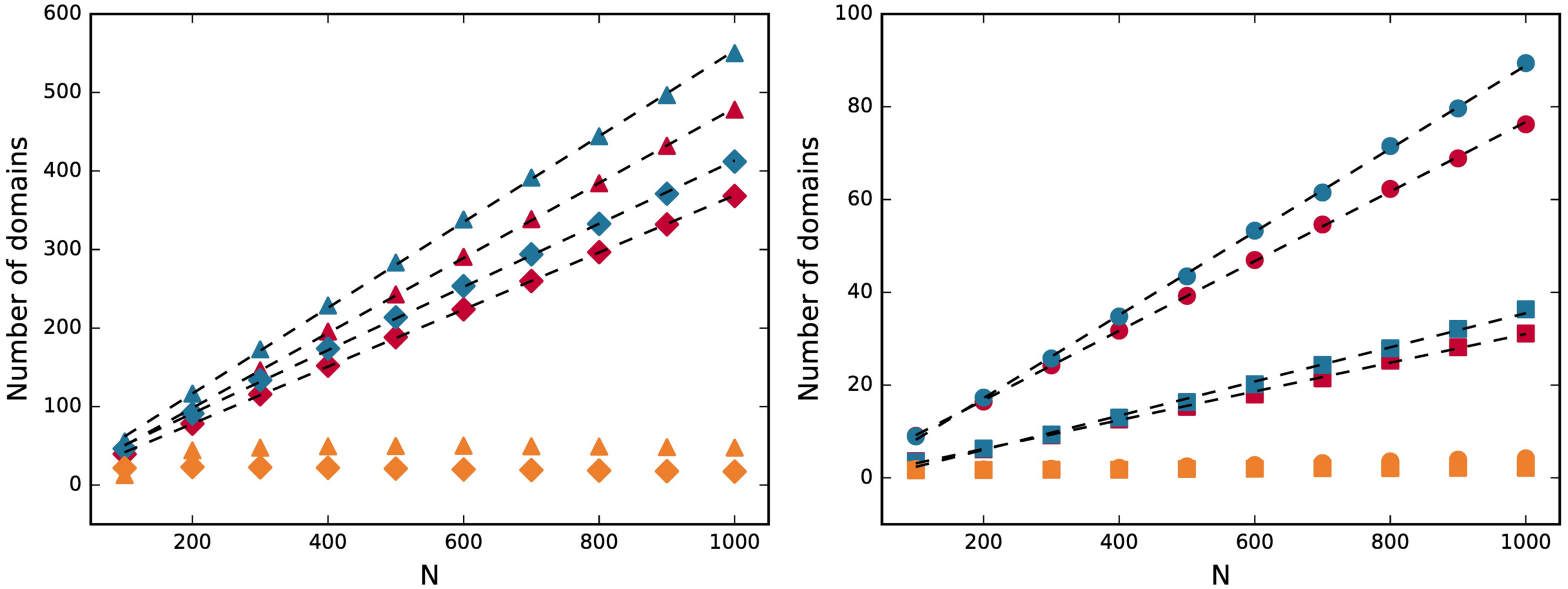
Number of domains as a function of the network size *N*. For the uniform model (red), the model with preferential attachment (blue), and the model form [19] (orange), for 〈*k*〉 = 4, *F* = 3, *q* = 2 (squares), *q* = 5 (circles), *q* = 50 (diamonds), and *q* = 100 (triangles), averaged over 400 realizations. Dashed lines represent the best linear fits.

In Fig 3 we analyze behavior of two variants of the model—local rewiring with a uniform probability and local rewiring with a preferential attachment. It is clear that the number of domains, indicating number of languages, increases with the system size linearly. Slope of the line strongly varies with the parameter *q*. For the plotted examples the slope coefficient of linear fits lays in a range from 3.099 · 10^−2^ ± 0.040 · 10^−2^ to 5.463 · 10^−1^ ± 0.037 · 10^−1^. For every fit the value of coefficient of determination is *R*^2^ > 0.99. This result is qualitatively consistent with the empirical data for Solomon Islands given in [52]. It is worth noting that this dependency is also valid, yet weaker, for different models described in [17], but only for a certain range of values of the parameter *q*. Note, that the number of domains for a fixed system size strongly depends on the parameter *q*. By varying its value we can adjust the number of languages to a particular empirical case. Additionally, as stated in the first work [18], one node can represent not only one person, but also a group of people. This means that the group must be homogeneous inside with everyone using exactly the same language, since everyone inside is described by the same vector of traits. This interpretation gives more flexibility in comparison with empirical data, as the size of the group is an additional parameter.

**Fig 4:**
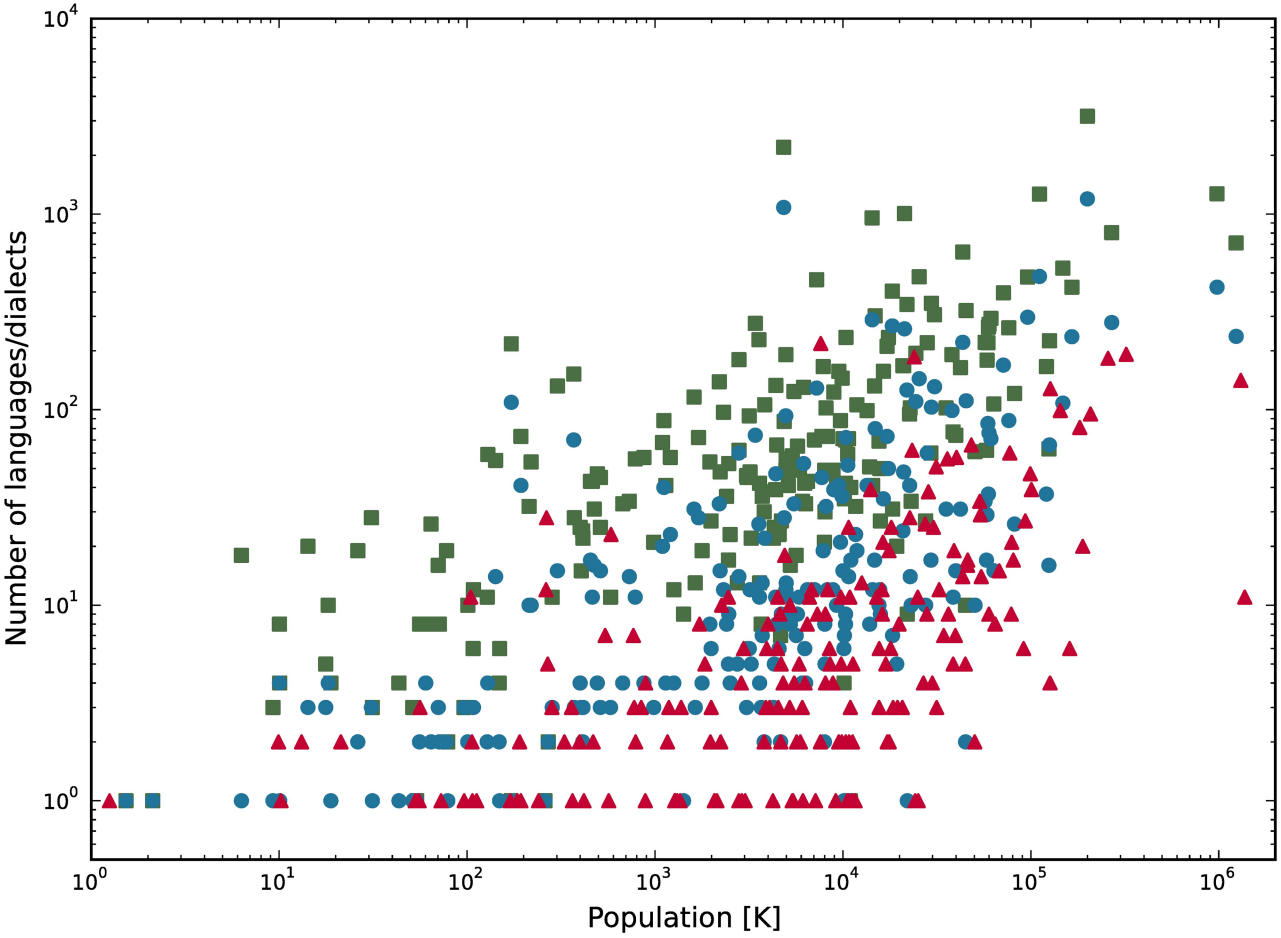
Empirical dependence of the number of languages on the size of a population. Red triangles represent languages from [54] and population sizes for 2015 [57]. Blue circles represent languages from [53] and population sizes for 1996 [57], green squares represent all dialects from the latter source.

**Fig 5:**
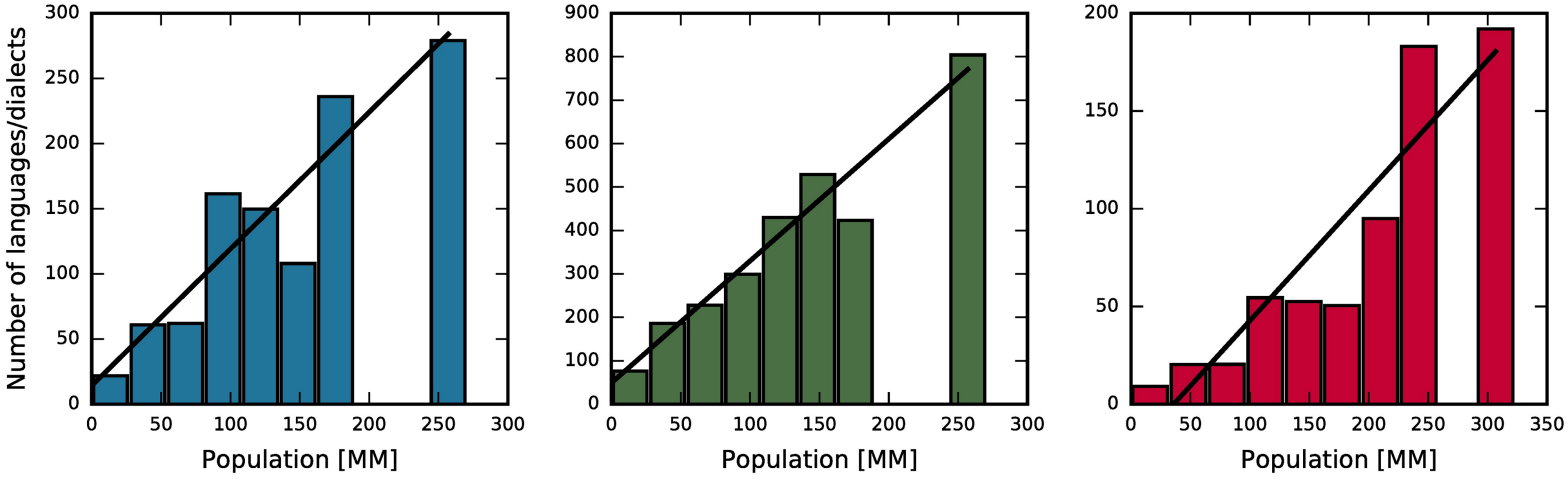
Dependence of the number of languages on the size of a population. Aggregated data from Fig 4, colors preserved. Height of a bar indicates the average number of languages in countries with a population lying within the bar. China, India, Indonesia, and Papua New Guinea are excluded. Solid lines represent linear fits with slope coefficients equal (from left to right) 1.05 ± 0.18, 2.81 ± 0.29, and 0.67 ± 0.11.

Based on our findings, we should expect larger number of languages for countries with bigger population. To validate this prediction we analyze two databases. The first one from 1996 consisting information about 6866 languages and their 9130 dialects from 209 different countries [53], and the second one from 2013 (regularly updated) consisting information about 2679 languages in 188 countries [54]. As we stated in the introduction, we do not intend to discuss subtle differences between languages and dialects. We take the data as given in the sources, and leave the judgment on how accurate is the distinction between languages and dialects for a curious readers. In Fig 4 we plot the number of languages against the size of a population for countries from six continents. The trend seems to be increasing in every example, but fluctuations darken the picture. Obviously, language diversity on a scale of continents is driven not only by social interactions. There are many factors influencing the linguistic structure of the society, for example language policy and legislation, colonization, border changes, demolition of the population during wars or epidemics, compulsory resettlement etc. Furthermore, nowadays television, radio and especially social media have a huge impact on the language we speak [39, 55, 56]. Nevertheless, we expect our findings to hold on average. To eliminate fluctuations we aggregate data for consecutive intervals. Results are shown in Fig 5, excluding, for the sake of clarity, four countries that have either the population size (China, India) or the number of languages (Indonesia, Papua New Guinea) grater by almost order of magnitude from the others. We obtain growing number of languages with the population size for both databases. Moreover, this dependency is even more pronounced in the data set of dialects. Again, results of the simulations are qualitatively consistent with the empirical data, what is an important result in context of previous models of this type, displaying behavior contradictory to empirical data.

In Fig 5 we also present on top of the aggregated data the best linear fits. For language data in both sources the coefficient of determination for the fit is equal *R*^2^ = 0.85, and for the data on dialects it is *R*^2^ = 0.94. Therefore, linear approximation describes the empirical data fairly well. Obviously, the data on dialects is the one most accurately described by the model, since the linear behavior is here most probable. Particular value of the slope coefficient can be adjusted by changing parameter *q* of the model and the size of the group of people represented by one node in the network. To get the idea about the order of magnitude of this size, we shall describe one example. The model for local rewiring with preferential attachment and parameters *F* = 3, *q* = 100 has the number of domains growing as 0.5463 ± 0.0037 of the number of nodes. Assume that this is a proper description of the empirical data on languages in [54] (red color in Fig 5). It is a plausible assumption according to 3*σ* rule. The data is described by a slope coefficient equal 0.67 ± 0.11, but with millions of people on the x-axis. This indicates that the model describes the empirical data, if one node stands for ∼10^6^ people.

## Conclusion

In our study we managed to cancel the contradiction between Axelrod-like models and empirical data on number of languages scaling with population size. We showed that even complex description of nodes’ states in social networks is not sufficient to explain real-world phenomena, if the influence of the structure of the network is not taken into account. Furthermore, even sophisticated dynamics of states can be not enough when the topology of the network is divergent to empirical examples. Topology and its transformations are crucial in the proper description of the language change due to social interactions. By changing the rewiring mechanism a model can convert from a contradiction to an agreement with real data. According to our results, models with local rewiring (triadic closure), leading to high clustering and frequent disintegration, most accurately reproduce empirical data. We believe it is an important result, pointing the direction for others simulating language change with complex networks. The model was designed as simple as possible to solve the scaling issue. There are, however, potential extensions giving perspectives for future work. For example, it would be interesting to study a model with different distributions of values for different language traits. Also, taking into account creation of new words and disappearance of old ones could enrich the model.

Here we have taken steps towards understanding how much of the process of the language change can be described by simple network models, not including all the relevant aspects, like geographical distribution or media influence. Full description of this subject is a highly complex problem, that goes beyond the scope of this paper. Nevertheless, comprehensive model of language should take into account appropriate dynamics of the network structure and our work suggests a proper direction in this matter.

## Materials and methods

### Algorithm

The model we use is based on the one described in [17]. We start every simulation with a random graph with *N* vertices, each representing one agent. We set the number of links *M* to obtain a certain value of the average degree 〈*k*〉. Every node *i* is described by a vector of traits *σ*_*i*_ = (*σ*_*i*,1_, *σ*_*i*,2_, …, *σ*_*i*,*F*_). Every trait can initially adopt one of *q* discrete values *σ*_*i*, *f*_ ∈ {1, 2, …, *q*}, *f* = 1, 2, …, *F*, what gives *q*^*F*^ possible different states. At the beginning, we draw a set of *F* traits for each node with equal probability for every value form 1 to *q*. Then, every time step consists of following rules:

1. Draw an active node *i* and one of its neighbors *j*.
2. Compare vectors *σ* of chosen vertices and determine the number *m* of identical traits (overlap), such that *σ*_*i*,*f*_ = *σ*_*j*,*f*_.
  - If all traits are equal i. e. *m* = *F*, nothing happens.
  - If none of the traits are equal *m* = 0, disconnect the edge (*i*, *j*) from node *j*, draw a new node *l*, and attach a link to it, creating an edge (*i*, *l*).
  - In other cases, with probability equal *m*/*F* the positive interaction occurs, in which we randomly select one of not-shared traits *f*′ (from among *F* − *m*) and the active node *i* adopts its value from the node *j*, i. e. $\sigma_{i,f^{\prime }}\to \sigma_{i,f^{\prime }}^{\prime }=\sigma_{j,f^{\prime }}$.
3. Go to the next time step.

The method of selecting new neighbors is crucial. We allow to create a new connection only within a set of nodes distant by two edges (neighbors of neighbors). Multiple connections and auto-connections are prohibited. We analyze two possibilities: uniform probability for every node in the set, and preferential attachment with probability *P*(*i*)∼(*k*_*i*_ + 1)^2^. Simulation is ran until frozen configuration is obtained or thermalization is reached. In order to describe behavior of the system we use several quantities and coefficients, which are defined as follows.

**Component *s***: two vertices *i* and *j* belong to the same component *s*, if they are connected, or vertex *k* exists such that vertex *i* belongs to the same component as vertex *k* and vertex *k* belongs to the same component as vertex *j*. Then, by the largest component of the network we mean the biggest connected subgraph of the network.

**Domain *d***: two vertices *i* and *j* belong to the same domain *d*, if they are connected and share all traits *σ*_*i*_ = *σ*_*j*_, or vertex *k* exists such that vertex *i* belongs to the same domain as vertex *k* and vertex *k* belongs to the same domain as vertex *j*. By definition, a given domain cannot exceed the size of the component it shares nodes with. On the other hand, the number of components cannot be superior to the number of domains.

**Local clustering coefficient *c*_*i*_**: for undirected graphs it can be defined as the number of connections between neighbors of the node *i* divided by *k*_*i*_(*k*_*i*_ − 1)/2, i.e. the number of links that could possibly exist between them.

**Global clustering coefficient *C***: it is defined as three times the number of triangles in the network divided by the number of connected triplets of vertices (one triangle consists three connected triplets).

**Average path length 〈*l*〉**: it is the shortest distance between two vertices, averaged over all pairs of vertices in the network. If there is no path between two vertices (network has many components), this pair is not taken into account.
